# Navigating Fertility Challenges: A Comprehensive Review of Microsurgery for Fallopian Tubal Re-canalization in the Era of In Vitro Fertilization (IVF)

**DOI:** 10.7759/cureus.54950

**Published:** 2024-02-26

**Authors:** Neha Sethi, Manjusha Agrawal, Deepika Dewani, Lucky Srivani Reddy, Anubha Dande

**Affiliations:** 1 Obstetrics and Gynaecology, Jawaharlal Nehru Medical College, Datta Meghe Institute of Higher Education & Research, Wardha, IND

**Keywords:** patient counseling, in vitro fertilization, reproductive technology, fertility treatment, microsurgery, tubal re-canalization

## Abstract

The journey of addressing fertility challenges, especially in the context of fallopian tubal issues, has witnessed remarkable advancements. This comprehensive review delves into microsurgery for fallopian tubal re-canalization within the era of in vitro fertilization (IVF). The review begins by providing the historical backdrop, tracing the evolution of this surgical technique, and highlighting the transformative impact of microsurgical methods and instrumentation on its precision and outcomes. Microsurgery for fallopian tubal re-canalization is characterized by a range of patient-specific considerations, diagnostic modalities, and factors influencing the choice between re-canalization and IVF. Microsurgical techniques are elaborated upon, showcasing the importance of laparoscopy and hysteroscopy, which not only diagnose but also treat tubal and uterine conditions. The review delves into the pivotal elements that steer the decision-making process, including patient preferences, medical necessity, ethical and religious considerations, financial constraints, and clinical evaluation. Furthermore, the intricate complications and risks associated with tubal re-canalization, both intraoperative and postoperative, are elucidated. Insights into recent advances in microsurgery, emerging research, and promising future directions set the stage for innovative and effective solutions. Recognizing the significance of patient counseling, shared decision-making, ethical considerations, and informed consent, the review underlines the critical role of healthcare providers in guiding individuals and couples through the complexities of fertility treatment choices. Finally, the conclusion synthesizes the key findings, implications for clinical practice, and future research directions, emphasizing the importance of tailored and patient-centered approaches to address fertility challenges in the modern era.

## Introduction and background

The quest for parenthood is a profoundly cherished aspiration for countless individuals and couples worldwide. However, for some, the journey to achieve this dream can be fraught with challenges, mainly when fertility issues come into play [1]. Fallopian tube obstructions are one such challenge, often hindering the natural conception process. While the introduction of assisted reproductive technologies (ART), notably in vitro fertilization (IVF), has revolutionized the field of reproductive medicine, there remains a steadfast interest in evaluating and refining alternative fertility restoration options. Among these alternatives, the fallopian tubal re-canalization microsurgical technique has garnered significant attention [1].

Fallopian tubal re-canalization, or tubal microsurgery, involves the meticulous surgical repair and restoration of fallopian tubes damaged or blocked due to various factors such as infections, adhesions, or prior surgeries [2]. This procedure seeks to reinstate the natural reproductive process, allowing the fertilization of an egg and the subsequent development of an embryo within the fallopian tubes. Given the continued prominence of tubal factor infertility and the availability of IVF as a popular alternative, this comprehensive review delves into the world of microsurgery for fallopian tubal re-canalization in the contemporary era of IVF [2].

The significance of this topic lies in the intersection of technological advancements, medical innovation, and patient-centric care in fertility treatment. While IVF has offered hope to many struggling with infertility, it is not always the most suitable or preferred option for every individual or couple. Tubal re-canalization represents an opportunity for those seeking a less invasive and more physiologic approach to conception, aligning with their values and preferences [3]. Moreover, the topic's significance is underscored by the nuanced decisions and considerations that patients and healthcare providers must make when choosing between IVF and tubal re-canalization. Understanding these approaches' advantages, disadvantages, and outcomes is crucial for ensuring informed, patient-centered care [4].

The primary purpose of this comprehensive review is to provide a comprehensive and up-to-date exploration of microsurgery for fallopian tubal re-canalization in the context of IVF. To fulfil this purpose, we have set the following objectives: Firstly, we aim to elucidate fallopian tubes' anatomical and functional aspects and their role in fertility, thereby establishing a foundational understanding of the subject matter. Secondly, we intend to offer a detailed overview of ART and IVF, emphasizing their advantages and limitations as fertility treatments. Thirdly, our review comprehensively examines microsurgery for fallopian tubal re-canalization, encompassing its historical development, surgical techniques, outcomes, and patient selection criteria. In addition, we delve into the diagnostic modalities used for tubal assessment, enabling a comprehensive evaluation of tubal health. Finally, we aim to explore the factors influencing the choice between tubal re-canalization and IVF, including patient-specific considerations, cost factors, ethical dilemmas, and clinical guidelines, to provide a well-rounded perspective on these fertility treatment options.

## Review

ART and IVF

Brief Overview of IVF as a Fertility Treatment

Ovarian stimulation: The first stage of the IVF process involves ovarian stimulation, a crucial step in maximizing the chances of a successful outcome. During this phase, a woman typically receives hormone medications that stimulate her ovaries to produce multiple follicles, each containing an immature egg [4,5]. This controlled ovarian hyperstimulation is essential because it allows for the development of several eggs in a single cycle, significantly increasing the likelihood of successful fertilization and embryo development. Monitoring and adjusting the hormone dosages are essential to ensure the follicles reach an optimal size before proceeding to the next step [5].

Egg retrieval: Once the follicles have matured, a minimally invasive transvaginal ultrasound-guided egg retrieval is performed. This step is critical for collecting the mature eggs without the need for major surgery. A specialized ultrasound probe is inserted into the vagina, guiding a thin needle into each mature follicle, where the eggs are gently aspirated. This process is usually conducted under sedation or anesthesia to ensure patient comfort. The retrieved eggs are then placed in a sterile environment and kept at an appropriate temperature until fertilization [6].

Fertilization: The next phase involves fertilizing the retrieved eggs with sperm, typically within a laboratory setting. There are two standard methods for achieving fertilization: conventional insemination and intracytoplasmic sperm injection (ICSI). In conventional insemination, sperm are combined with the eggs in a dish, and natural fertilization occurs as the sperm penetrates the egg's outer layer. In contrast, ICSI is used in cases of male infertility or when conventional insemination is unlikely to be successful. With ICSI, a single sperm is directly injected into the egg, ensuring fertilization. Both methods aim to create embryos, which will be cultured in a controlled environment for a few days before being transferred to the woman's uterus or frozen for future use, depending on the specific circumstances and the patient's reproductive goals [7].

Embryo culture: After fertilization, the resulting embryos are carefully cultured in a controlled laboratory environment for several days, usually up to five. The embryos progress through various developmental stages during this crucial period, and their quality is assessed. This culture period allows embryologists to monitor their growth and development, ensuring that they are healthy and have the potential to result in a successful pregnancy. The embryos are typically kept in incubators with precise temperature, humidity, and gas concentration controls to mimic the conditions within the female reproductive tract [8].

Embryo transfer: The next step in the IVF process is the selection and transfer of one or more healthy embryos into the woman's uterus. The timing of this transfer is usually determined based on the embryos' development, commonly occurring on either day 3 or day 5 post-fertilization. The choice between these options may depend on the number and quality of embryos, the patient's age, and the clinic's protocols. During the embryo transfer procedure, a thin catheter is inserted through the cervix into the uterus, and the selected embryos are carefully placed in the ideal location for implantation [9].

Luteal-phase support: To support the successful implantation of the embryos, hormonal medications may be prescribed to ensure that the uterine lining is receptive and conducive to embryo attachment. The luteal phase is the second half of the menstrual cycle, and these medications help maintain a favourable hormonal environment for pregnancy. This phase support may include progesterone supplements and other medications to support the uterine lining, ultimately enhancing the chances of successful embryo implantation and a healthy pregnancy [10].

Pregnancy testing: Approximately two weeks after the embryo transfer, a pregnancy test is conducted to determine whether the IVF procedure has been successful. This test measures the human chorionic gonadotropin (hCG) level in the woman's blood or urine. Elevated levels of hCG indicate a successful pregnancy, and a positive test result is an exciting milestone for hopeful parents. The outcome of this test provides valuable information about the success of the IVF cycle and marks the beginning of the pregnancy journey for those who receive positive results [11].

Advantages and Limitations of IVF

High success rates: IVF is known for its relatively high success rates, making it a valuable option for many couples struggling with infertility. The success rates can vary depending on factors such as the age of the female partner, the underlying cause of infertility, and the quality of the embryos, but, on average, IVF offers a reasonable chance of achieving a successful pregnancy. This makes it a promising choice for those who have not had success with other fertility treatments or who face complex fertility issues [12].

Addressing various causes: IVF is a versatile fertility treatment that can effectively address a wide range of infertility factors. It is particularly beneficial for couples dealing with male factor infertility, tubal factor infertility, or unexplained infertility. For example, in cases of male factor infertility where the sperm quality or quantity is compromised, IVF can bypass these issues by directly injecting sperm into the egg through ICSI. For couples with tubal factor infertility due to damaged or blocked fallopian tubes, IVF allows for fertilization to occur in the laboratory, completely circumventing the need for functional fallopian tubes [13].

Genetic testing: IVF offers the option of preimplantation genetic testing (PGT), a powerful tool to screen embryos for genetic disorders before implantation. This is particularly valuable for couples who risk passing genetic conditions to their offspring. PGT allows the selection of embryos free of known genetic abnormalities, reducing the risk of genetic diseases in the resulting child. It also enables the selection of embryos of a preferred sex in cases where gender-related genetic disorders are a concern [14].

Donor options: IVF provides the flexibility to use donor eggs or sperm, allowing couples and individuals to build a family when they are unable to use their gametes. This is especially helpful for those facing severe male or female infertility, as well as same-sex couples or single individuals who require donor gametes to achieve pregnancy. Donor eggs and sperm can be carefully selected to match the desired characteristics of the intended parents, allowing them to have a genetically related child even when they cannot provide their genetic material. This inclusivity and diversity in family-building options are a significant advantage of IVF [15].

Cost: One of the significant factors that individuals and couples must consider when contemplating IVF is the financial burden it can impose. IVF can be quite expensive, with costs encompassing various stages, including ovarian stimulation, egg retrieval, embryo culture, and embryo transfer. The need for multiple cycles to achieve a successful pregnancy can significantly escalate these expenses. These costs may not be covered by insurance, leaving patients to bear the financial responsibility, which can create stress and financial strain [16].

Emotional and physical demands: IVF is not just a medical procedure but also an emotional journey that can be taxing for those undergoing it. The process often involves daily hormone injections, frequent medical appointments, and the emotional rollercoaster of hope and uncertainty. The emotional demands can be incredibly challenging as individuals and couples grapple with the pressure, anxiety, and potential disappointment of failed cycles. The physical demands, including the side effects of hormonal treatments, can be uncomfortable and fatiguing. Balancing these emotional and physical demands while maintaining daily life and work commitments can be extremely challenging [17].

Multiple pregnancies: A potential consequence of IVF is the increased risk of multiple pregnancies, including twins or triplets. While some may view this as a positive outcome, it carries certain risks and challenges. Multiple pregnancies can lead to a higher likelihood of premature birth, low birth weight, and other health complications for both the mother and the babies. It may necessitate more intensive medical care and monitoring, adding further stress to the already demanding IVF journey [18].

Not suitable for all: IVF may not be the best option for everyone dealing with infertility. The effectiveness of IVF can be influenced by various factors, such as the age and overall health of the patient, the cause of infertility, and the quality of eggs and sperm. Sometimes, individuals or couples may not be good candidates for IVF due to medical conditions, financial constraints, or personal preferences. Healthcare providers need to consider individual circumstances and discuss alternative fertility treatments when IVF may not be the best course of action [19].

Indications for Tubal Re-canalization in the IVF Era

Patient preference: One of the primary factors influencing the choice between tubal re-canalization and IVF is patient preference. Some individuals and couples may strongly desire a more natural conception process. This preference often becomes especially relevant when the cause of infertility is primarily related to tubal factors. Patients may prioritize the idea of achieving pregnancy without the need for ART, making tubal re-canalization an appealing option [2].

Medical necessity: In cases where tubal obstructions or damage can be effectively addressed through re-canalization, it may be the recommended and necessary treatment. Healthcare providers may evaluate the specific nature of the tubal issue and determine that surgical intervention is the most appropriate course of action to restore fertility. This medical necessity can be a crucial factor in guiding the decision-making process [20].

Ethical or religious considerations: Ethical and religious beliefs can play a significant role in the choice between tubal re-canalization and IVF. Some patients may have personal or cultural reasons to avoid IVF, often due to concerns related to the handling of embryos or the use of ART. These ethical or religious considerations can strongly influence the decision favouring tubal re-canalization [21].

Cost considerations: Financial constraints can be a substantial factor in the decision-making process. IVF can be costly, with expenses often associated with multiple cycles and various stages of the procedure. When the financial burden is a significant concern, tubal re-canalization is a more cost-effective option. This is particularly relevant for patients without adequate insurance coverage for fertility treatments [22].

Clinical evaluation: The recommendation for tubal re-canalization versus IVF is also based on a thorough clinical evaluation by healthcare providers. Factors such as the patient's overall health, tubal damage or obstructions, and the likelihood of successful re-canalization are all assessed. Healthcare providers consider the patient's specific circumstances and medical history to make recommendations that align with their needs [23].

Microsurgery for fallopian tubal re-canalization

Historical Perspective and Evolution

Historical background: The history of fallopian tubal re-canalization dates back to the mid-20th century, marking a significant turning point in fertility treatment. Surgeons began to explore methods to repair and restore damaged fallopian tubes as a means to address tubal factor infertility. During this era, the techniques used were relatively rudimentary and often required laparotomy, a more invasive surgical procedure involving a large abdominal incision. Early methods relied on various microsuturing techniques to reopen or repair the damaged tubes. While these pioneering efforts represented a vital step in developing tubal re-canalization, they were associated with greater invasiveness, longer recovery times, and higher risks [24].

Advances in microsurgery: The latter part of the 20th century witnessed significant advances in microsurgery that profoundly transformed the field of fallopian tubal re-canalization. The introduction and widespread adoption of advanced microsurgical techniques and instrumentation characterized this period. The cornerstone of these advances was the utilization of powerful microscopes and specialized instruments that provided surgeons unprecedented precision and control during surgical procedures. The integration of these technological innovations enabled more delicate and refined interventions, ultimately minimizing tissue damage and scarring [25]. By significantly reducing the invasiveness of the procedure, microsurgery brought about a paradigm shift in the field, making tubal re-canalization an effective and minimally invasive option for addressing tubal infertility. Patients could now benefit from surgical interventions that improved the chances of tubal patency and reduced postoperative discomfort, recovery times, and potential complications, thereby enhancing their overall experience and outcomes. These advances in microsurgery continue to play a vital role in modern fertility treatment, offering a gentler and more precise approach to restoring fertility in individuals with tubal factor infertility [25].

Surgical Techniques and Instrumentation: Microsurgical Techniques

Tubal re-canalization is a highly specialized procedure that relies on microsurgical techniques to restore tubal patency and address tubal factor infertility. These techniques involve the following steps:

Laparoscopy: The procedure typically begins with laparoscopy, a minimally invasive surgical technique. A small incision is made near the navel, and a laparoscope, a thin, lighted tube with a camera, is inserted into the abdominal cavity. The laparoscope provides real-time imaging of the fallopian tubes and the surrounding reproductive structures. This precise visualization allows the surgeon to assess the condition of the fallopian tubes, identify any obstructions, damage, or other issues, and plan the re-canalization procedure accordingly [25].

Microsurgical instruments: Microsurgery relies on specialized instruments for delicate and precise manipulations. These instruments are much smaller than conventional surgery ones and include microscissors, microforceps, and micro-needle holders. These microsurgical instruments enable the surgeon to work with exceptional precision, handling the fine and delicate structures of the fallopian tubes with great care. This level of precision minimizes tissue trauma and scarring, which is crucial for optimizing postoperative outcomes [26].

Tubal evaluation: The fallopian tubes are meticulously evaluated before proceeding with any corrective measures. The surgeon carefully examines the tubes to determine the exact location and nature of the blockage or damage. This evaluation is critical in tailoring the re-canalization procedure to the individual patient's needs. It ensures that the surgeon's interventions are targeted and effective in addressing the tubal factors contributing to infertility [27].

Tubal re-canalization: After the initial assessment, the surgeon proceeds with tubal re-canalization. This may involve the use of microsuturing techniques or other microsurgical methods. Microsuturing is an exact technique in which the surgeon delicately sews or repairs the tubal structures to remove obstructions, mend damage, or create a new passage within the fallopian tubes. The goal is to re-establish unobstructed patency, allowing the eggs to travel from the ovaries to the uterus, where fertilization can occur naturally. Microsurgical instruments ensure that these repairs are performed accurately, minimizing tissue trauma and preserving the delicate anatomy of the fallopian tubes [4].

Intravascular stenting: Intravascular stenting is a technique that may be employed in some instances to help maintain the patency of the fallopian tubes after tubal re-canalization surgery. This procedure involves the placement of a stent, a tiny, tubular device made of biocompatible materials, within the fallopian tubes. The stent serves as a scaffold to keep the tubes open and unobstructed, facilitating the passage of eggs from the ovaries to the uterus [28].

The use of intravascular stenting is typically considered in situations where there is a higher risk of postoperative scarring, restenosis (re-narrowing of the tubes), or recurrent blockages. By deploying a stent, surgeons aim to provide ongoing structural support to the fallopian tubes, preventing them from collapsing or closing again. The placement of intravascular stents is typically performed as part of the tubal re-canalization procedure or as a follow-up step immediately after the surgical correction. It is a minimally invasive process, often carried out using imaging guidance to ensure accurate stent positioning [29].

The decision to use intravascular stenting depends on individual patient factors, such as the extent of tubal damage, the likelihood of postoperative issues, and the surgeon's assessment of the patient's specific needs. Using stents can enhance the long-term success of tubal re-canalization and may contribute to more sustained fertility outcomes. Patients should discuss the potential benefits and risks of intravascular stenting with their healthcare providers to make informed decisions regarding their fertility treatment [20].

Success Rates and Outcomes

Success rates: Success rates of tubal re-canalization through microsurgery can vary depending on the patient's circumstances. In general, microsurgery has demonstrated promising outcomes in re-establishing tubal patency. The success rates tend to be higher for patients with simpler tubal blockages, such as minor obstructions, compared to those with more complex tubal issues, such as extensive damage or severe scarring. The procedure's success depends on the extent of tubal destruction and the length of the repaired tubal segments. If the reconstructed tube has a length of at least 4 cm with an ampullary length of 1 cm, live birth rates of 60-80% can be achieved. The tubal pregnancy rates vary but average 2-5% [30]. The likelihood of success is often assessed based on factors like the location and nature of the blockage and the patient's overall health and fertility status [30].

Pregnancy rates: Successful tubal re-canalization can lead to natural pregnancies. The chances of achieving a successful pregnancy post-surgery depend on multiple factors. A critical factor is the woman's age, as fertility declines with age. Younger patients generally have a higher likelihood of conceiving naturally after the procedure. Additionally, the presence of any other fertility issues, in both the female and male partner, can influence pregnancy rates. Ovulatory disorders, sperm quality, and other factors need to be considered. Couples should engage in regular unprotected intercourse following the surgery to optimize the chances of conception [4].

Minimized risks: Microsurgery offers several advantages in minimizing the risks associated with the procedure. Unlike traditional open surgery, microsurgery is minimally invasive, typically performed through small incisions with specialized microsurgical instruments. This minimizes the risk of complications, such as adhesions (scar tissue formation) and extensive scarring, which can hinder tubal function. Patients often experience a quicker recovery and reduced postoperative pain, allowing them to return to their daily activities more rapidly [31].

Follow-up: Postoperative care and follow-up are critical aspects of the tubal re-canalization process. After the procedure, patients are typically closely monitored to assess the effectiveness of the surgery and ensure that the fallopian tubes remain open and functional. Some patients may require additional interventions or fertility treatments to enhance their chances of conception. Follow-up care also includes assessing the outcomes of pregnancies achieved after the procedure, as there may be a slightly higher risk of ectopic pregnancies, which require prompt diagnosis and management [32].

Patient Selection Criteria

Age: Age plays a critical role in the success of tubal re-canalization. Younger patients have better outcomes due to their eggs' higher quality and quantity. Fertility declines with age, and as women get older, the success of tubal re-canalization and the likelihood of achieving a pregnancy may decrease. Younger patients typically have a more favourable prognosis for natural conception after the procedure [32].

Type of tubal damage: As identified through diagnostic tests, the nature and extent of tubal damage significantly influence re-canalization success. More straightforward tubal issues, such as minor blockages or mild scarring, are more amenable to successful repair. However, more complex problems like extensive damage or severe scarring may be less likely to respond well to surgical intervention. The location and severity of the damage are essential considerations in the decision-making process [33].

Overall health: The patient's health and medical history are essential to assessing their suitability for undergoing tubal re-canalization surgery. Certain medical conditions or complications could increase the risks associated with surgery or interfere with postoperative recovery. A thorough evaluation of the patient's health status ensures they are physically prepared for the procedure and minimizes the potential for complications [34].

Patient preferences: Patient preferences are a central component of the decision-making process. The choice between tubal re-canalization and alternative fertility treatments, such as IVF, often depends on the patient's willingness to undergo a surgical procedure. Some individuals and couples have a strong preference for natural conception and may prioritize tubal re-canalization, while others may be more open to IVF or other ART. Personal values, experiences, and perceptions of the treatment process influence these preferences [13].

Financial considerations: Financial considerations also play a significant role in decision-making. The cost of tubal re-canalization, including surgical fees, diagnostic tests, and associated medical expenses, can be critical. Patients may need to consider their ability to afford the procedure and the availability of insurance coverage for fertility treatments. Financial constraints can influence the choice between tubal re-canalization and alternative treatments like IVF, which may have different cost structures [4].

Diagnostic modalities for tubal assessment

Hysterosalpingography (HSG) 

HSG is a critical diagnostic procedure used in the realm of reproductive medicine to assess the condition and patency of the fallopian tubes, playing a pivotal role in the evaluation of female fertility. The procedure involves the injection of a radiopaque contrast dye into the uterine cavity, typically carried out in a radiology suite or clinic setting. This contrast dye is carefully introduced using a thin catheter guided through the cervix. This dye is "radiopaque" because it is visible on X-ray images. Subsequently, a series of real-time X-ray images are captured to track the flow of the contrast dye as it moves through the fallopian tubes. These images allow radiologists and healthcare professionals to observe whether the dye flows unobstructed through the tubes and into the abdominal cavity [35].

HSG offers numerous advantages as a diagnostic tool for evaluating tubal patency. It is minimally invasive and is generally well-tolerated by patients. The real-time X-ray images provide immediate visual confirmation of the status of the fallopian tubes, indicating whether they are open and free from blockages. HSG is often the initial diagnostic test employed to assess tubal patency, and its results can guide healthcare providers in determining the most suitable treatment approach for individuals experiencing infertility. The procedure is relatively quick and does not necessitate sedation or anesthesia, making it accessible and convenient for many patients [36].

Nevertheless, HSG does have certain limitations. Its primary focus is on evaluating tubal patency and may not offer a comprehensive assessment of the structural integrity of the fallopian tubes. While it can detect major blockages or obstructions, it may not always reveal subtle tubal issues, such as minor adhesions or mucosal irregularities. Moreover, HSG does not assess the functional capabilities of the fallopian tubes, such as their ability to transport eggs and embryos effectively. Therefore, additional evaluation or other diagnostic tests may be necessary in some cases to gain a more comprehensive understanding of tubal health. Despite its limitations, HSG remains an invaluable tool in the diagnostic process for individuals seeking answers regarding their fertility and offers a vital first step in identifying and addressing tubal-related issues [37].

Saline Infusion Sonohysterography (SIS)

SIS is a diagnostic technique that plays a crucial role in evaluating the uterine cavity and has some relevance in assessing the fallopian tubes. The procedure involves the careful combination of a transvaginal ultrasound and the introduction of a sterile saline solution into the uterine cavity. This technique is typically conducted in a clinical or radiology setting, offering several key advantages and limitations [38].

During an SIS, a thin catheter introduces a sterile saline solution into the uterine cavity. Simultaneously, a transvaginal ultrasound probe provides real-time visualization of the uterine and tubal structures. The saline infusion expands and defines the uterine cavity and the endometrial lining, enhancing the clarity and precision of the ultrasound images. This dynamic process enables the detection of abnormalities or irregularities within the uterine cavity and its lining [39].

The advantages of SIS are significant. It is a valuable tool for identifying various uterine abnormalities, such as polyps, fibroids, adhesions, or congenital anomalies. Additionally, it can indirectly detect conditions that might impact tubal function, like hydrosalpinx, where fluid accumulates within a fallopian tube. Recognizing these uterine and tubal issues is critical, as they can contribute to fertility problems or potential complications during pregnancy [40].

However, SIS does have limitations. There are more effective methods for evaluating tubal patency or directly visualizing the fallopian tubes' inner lumen. While it can identify specific tubal issues, such as hydrosalpinx, it does not directly assess the functional capabilities of the fallopian tubes. As a result, there may be better diagnostic tests than SIS for confirming the patency of the fallopian tubes, as other procedures like HSG offer a more direct evaluation of tubal patency. Instead, SIS primarily focuses on the uterine cavity and its associated abnormalities, making it a valuable but somewhat limited tool in the diagnostic toolkit for reproductive health and fertility assessment [41].

Laparoscopy and Hysteroscopy

Laparoscopy and hysteroscopy are advanced and minimally invasive surgical techniques crucial in diagnosing and treating various tubal and uterine conditions in gynecology and reproductive medicine. Laparoscopy involves the insertion of a laparoscope, a thin, lighted tube with a camera, through a small incision made in the abdominal wall. This procedure allows for the direct visualization of the pelvic organs, including the fallopian tubes, the ovaries, and the outer surface of the uterus. Hysteroscopy, on the other hand, utilizes a slender scope to examine the interior of the uterine cavity, providing an up-close view of the uterine lining [42].

One of the key advantages of these procedures is their ability to provide direct and precise visualization of the fallopian tubes, which is crucial for identifying and addressing a range of reproductive health issues. Both laparoscopy and hysteroscopy also offer the option for surgical interventions if necessary. For instance, they are highly effective in diagnosing and treating conditions such as adhesions, polyps, and endometriosis. The minimally invasive nature of these techniques results in shorter recovery times, smaller incisions, and reduced postoperative pain compared to traditional open surgeries [43].

However, it's important to note that laparoscopy and hysteroscopy have limitations. They require anesthesia, which carries its own risks and complications. These procedures are typically reserved for cases in which surgical intervention is indicated, such as tubal surgery to address issues like tubal blockages or uterine anomalies. In cases where less invasive treatments or fertility interventions can resolve the issue, healthcare providers may explore those options first [44].

Other Imaging and Diagnostic Tools

Magnetic resonance imaging (MRI): MRI is a powerful imaging technique that can provide highly detailed and cross-sectional images of the reproductive organs, including the uterus and fallopian tubes. It is beneficial for assessing structural abnormalities, such as uterine fibroids, congenital anomalies, or tubal pathology. MRI offers a non-invasive and comprehensive view of these organs, aiding in the diagnosis and treatment planning for various reproductive conditions [45].

Three-dimensional (3D) ultrasound: 3D ultrasound technology enhances the imaging of uterine and tubal structures. It provides 3D images that can be manipulated to visualize reproductive organs from multiple angles, diagnosing anomalies such as uterine polyps, fibroids, and tubal abnormalities. This non-invasive and real-time imaging tool is widely used in gynecology to assess reproductive health [46].

Sonohysterosalpingography (SHSG): SHSG is a diagnostic procedure that combines elements of both HSG and SIS. It involves using saline and contrast dye to simultaneously assess the conditions of the uterine cavity and fallopian tubes. This integrated approach allows for evaluating structural and patency issues in a single procedure, comprehensively assessing female reproductive health [47].

Serum biomarkers: Besides imaging techniques, blood tests may be employed to assess specific biomarkers associated with tubal and uterine health. One such biomarker is anti-Müllerian hormone (AMH), which can provide information about ovarian reserve and is sometimes used to indicate tubal issues. While these biomarkers may not directly visualize the tubal or uterine structures, they can offer valuable information about ovarian function and, indirectly, the reproductive potential of an individual [48].

Complications and risks associated with tubal re-canalization

Intraoperative Complications

Bleeding: There is a risk of unexpected bleeding during the surgery, although this risk is typically low. In the event of excessive bleeding, the surgical team must take immediate measures to control the bleeding, including suturing blood vessels or using hemostatic agents. It's worth noting that surgical procedures are conducted with the utmost care to minimize bleeding risks [49].

Infection: Surgical site infections are a potential complication of any surgery, including tubal re-canalization. To mitigate this risk, the surgical team follows strict sterile protocols, and patients are often prescribed prophylactic antibiotics before the procedure. If an infection does occur, it can be treated with appropriate antibiotics [50].

Injury to surrounding structures: There's a small risk of unintentional damage to adjacent organs or structures during the procedure. For example, during re-canalization, there may be a rare chance of injuring nearby blood vessels or the intestines. Additional surgical corrections may be needed to repair the damage in such an injury. It's important to remember that these instances are infrequent and surgical teams are highly trained to minimize such risks [51].

Difficulties in tubal access: Some cases may present challenges in gaining access to the fallopian tubes, which can extend the duration of the surgery. This may occur if anatomical variations or complexities make reaching and repairing the tubes more challenging. Extended surgical time can increase the risk of complications, so surgeons take measures to ensure safe and efficient procedures [52].

Postoperative Complications

Adhesions: The formation of scar tissue, known as adhesions, in the pelvic area is a possible complication of tubal re-canalization. Adhesions can develop as a result of the surgical procedure itself or due to pre-existing conditions. These adhesions may attach the fallopian tubes to nearby structures or organs, potentially causing further tubal blockages or other fertility issues. The presence of adhesions can make it difficult for the fallopian tubes to function properly, hindering the passage of eggs or embryos and impacting fertility [53].

Ectopic pregnancy: Ectopic pregnancy is a condition in which a fertilized egg implants and begins to develop outside the uterus, typically in one of the fallopian tubes. There is a slightly increased risk of ectopic pregnancies following tubal re-canalization. This is because the surgical procedure may not completely restore normal tubal function and there remains a risk of the fertilized egg becoming trapped in a partially blocked tube. Ectopic pregnancies are life-threatening and require immediate medical attention and intervention to prevent complications [54].

Infection: Like any surgical procedure, tubal re-canalization carries a risk of postoperative infections in the pelvic area. These infections can occur at the surgical site or within the reproductive organs. Infections may cause symptoms such as pain, fever, or abnormal discharge. Prompt medical attention and appropriate antibiotic treatment are necessary to manage and resolve these infections [55].

Persistent tubal issues: In some cases, tubal re-canalization may not fully resolve the underlying tubal problem, and the tubes may continue to experience issues such as recurrent blockages, adhesions, or other structural abnormalities. Despite the surgical intervention, fertility challenges may persist. If this occurs, patients and their healthcare providers may need to explore alternative fertility treatments or strategies to address ongoing fertility issues [56].

Long-Term Risks and Considerations

Tubal function: The long-term success of tubal re-canalization is closely linked to the nature and extent of the original tubal damage. The procedure aims to repair and restore the fallopian tubes to their normal function. However, there is a potential for re-blockage or the development of adhesions over time, which could affect tubal function. The durability of the repair and the prevention of further damage depend on several factors, including the underlying cause of the tubal issue and the quality of the surgical intervention [57].

Future fertility: Patients should think about their desire for future pregnancies when considering tubal re-canalization. The procedure's success may influence the potential for future pregnancies and their timing. Couples or individuals should communicate with their healthcare providers about the expected post-surgery fertility outlook, keeping in mind that achieving pregnancy post-re-canalization can take some time and multiple cycles may be needed [4].

Recurrent tubal issues: There is a risk of recurrent tubal problems even after successful tubal re-canalization. In some cases, the same or new issues with the fallopian tubes may arise, necessitating further treatments or fertility interventions. Patients should be prepared for the possibility of ongoing fertility challenges and the need for additional medical attention [2].

Pregnancy outcomes: Pregnancy outcomes following tubal re-canalization should be closely monitored. There may be an increased risk of certain pregnancy complications, such as ectopic pregnancies (where the fertilized egg implants outside the uterus), which can be life-threatening if not promptly detected and managed. Healthcare providers will closely follow pregnancies achieved after the procedure to ensure the best possible outcomes for both the mother and the developing fetus [58].

Alternative options: In cases where tubal re-canalization is not successful or fertility issues persist, it's essential to consider alternative fertility treatments. IVF is one such option that may be considered. Understanding the available alternatives and being prepared to explore them if needed is essential for individuals or couples seeking to build their families. IVF, for example, can offer a viable route to pregnancy when the fallopian tubes are a hindrance to natural conception [59].

Recent advances in microsurgery for tubal re-canalization

Innovative Techniques and Technologies

Robotic-assisted surgery: Robotic systems have revolutionized the field of surgery, and their application has extended to tubal re-canalization. Robotic-assisted laparoscopic surgery allows surgeons to perform intricate procedures with increased precision and dexterity. By using robotic arms, surgeons can manipulate instruments more effectively within the body, and the robotic system provides 3D visualization of the surgical field. This technology improves the surgeon's ability to navigate and repair tubal issues, ultimately enhancing success rates and reducing the procedure's invasiveness [60].

Laser-assisted procedures: Laser technology has been integrated into gynecology and reproductive medicine to address tubal issues more precisely. Laser-assisted procedures can open or repair tubal blockages with minimal damage to surrounding tissue. The focused energy of lasers can be precisely controlled, making them an excellent choice for delicate procedures like tubal re-canalization. Laser-assisted surgery offers the advantage of reduced scarring, shorter recovery times, and potentially improved fertility outcomes [61].

Fluoroscopy and 3D imaging: Advanced imaging techniques, including 3D fluoroscopy, have significantly enhanced the visualization of the fallopian tubes during tubal re-canalization procedures. Fluoroscopy provides real-time X-ray images that help surgeons precisely navigate and treat tubal issues. The addition of 3D imaging allows for a more comprehensive view of the tubal structures, aiding in the accurate diagnosis and treatment of blockages, adhesions, or other anomalies. This improved visualization enhances the overall safety and success of the procedure [62].

Intraoperative ultrasound: Intraoperative ultrasound is another valuable tool for surgeons performing tubal re-canalization. It allows for real-time imaging and assessment of tubal patency and the immediate impact of surgical interventions. This technology lets surgeons confirm that the fallopian tubes are properly reopened or repaired during the procedure, ensuring the intended results are achieved [63].

Emerging Research in the Field

Biomaterials and biodegradable stents: Researchers are exploring using biomaterials and biodegradable stents or scaffolds to maintain tubal patency post-surgery. These innovative materials can be placed in the fallopian tubes to prevent restenosis (re-blockage) after tubal re-canalization. Biodegradable stents gradually dissolve in the body, reducing the risk of complications associated with long-term foreign body presence. This research aims to enhance the long-term success of the procedure and improve fertility outcomes [64].

Innovations in imaging: Advances in imaging technology play a crucial role in diagnosing and assessing tubal patency. Researchers are investigating the utility of contrast-enhanced ultrasound and MRI for their ability to provide detailed and accurate information about the fallopian tubes. These technologies offer non-invasive alternatives to traditional imaging methods, potentially improving the precision and efficiency of diagnosis [65].

Genetic and molecular studies: Understanding the genetic and molecular factors that impact tubal health is a burgeoning area of research. Genetic and molecular studies shed light on the underlying causes of tubal issues, such as inflammation, fibrosis, or scarring. Identifying specific genetic markers and molecular pathways associated with tubal damage can lead to the development of targeted therapies to improve the success of re-canalization procedures [66].

Patient selection algorithms: Researchers are developing personalized algorithms that consider a patient's unique characteristics and diagnosis to determine the most suitable treatment option. These algorithms can help healthcare providers make more informed decisions about whether tubal re-canalization, IVF, or other fertility treatments are the best choice for a particular patient. Personalized treatment recommendations can optimize the chances of successful conception and pregnancy [67].

Promising Future Directions

Precision medicine: Precision medicine involves tailoring medical treatments to individual patients based on their genetic and clinical characteristics. Applying precision medicine to tubal re-canalization procedures may enhance success rates by customizing treatments for each patient's needs. By identifying genetic markers and other factors contributing to tubal issues, healthcare providers can develop more targeted and effective treatment plans [68].

Regenerative medicine: Researchers are exploring regenerative medicine approaches, such as stem cell therapies and tissue engineering, as potential solutions for repairing damaged fallopian tubes. These innovative techniques aim to restore tubal function by promoting tissue regeneration and repair, offering new possibilities for addressing tubal-related fertility challenges [69].

Enhanced patient counseling: Developing comprehensive decision-support tools and patient counseling approaches will empower individuals to make well-informed choices regarding fertility treatments. This includes providing precise and personalized information about the available options, potential outcomes, and associated risks, allowing patients to play an active role in their treatment decisions [70].

Quality-of-life considerations: Future research will increasingly focus on the psychological and quality-of-life aspects of patients undergoing tubal re-canalization and related procedures. Assessing and addressing individuals' emotional well-being and overall quality of life on their fertility journeys will be integral to providing holistic care and support [71].

Global access: Efforts to make advanced tubal re-canalization techniques more accessible and affordable globally will continue to evolve. Ensuring that individuals from diverse backgrounds and regions have access to these innovative treatments is a crucial goal. This may involve reducing costs, increasing training and infrastructure, and expanding the availability of these procedures in underserved areas [56].

## Conclusions

This comprehensive review has explored the multifaceted landscape of microsurgery for fallopian tubal re-canalization in the era of IVF. It has highlighted the historical evolution of this surgical technique, its innovative tools and technologies, and the ever-emerging research that propels its advancement. The review underscored the importance of patient-centered care, emphasizing patient education, shared decision-making, and ethical considerations in choosing between tubal re-canalization and IVF. Moreover, discussing diagnostic modalities, complications, and risks associated with the procedure informed patients and clinicians alike. Notably, recent breakthroughs in the field and promising future directions, such as regenerative therapies and global accessibility, offer hope for even more effective fertility solutions. As we move forward, the intersection of informed consent, personalized care, and cutting-edge technology will continue to shape the landscape of microsurgery for tubal re-canalization, ultimately providing individuals and couples with a more comprehensive and tailored approach to addressing fertility challenges.
